# Measuring quality of dying, death and end-of-life care for children and young people: A scoping review of available tools

**DOI:** 10.1177/02692163221105599

**Published:** 2022-08-01

**Authors:** Catriona R Mayland, Katy A Sunderland, Matthew Cooper, Paul Taylor, Philip A Powell, Lucy Zeigler, Vicki Cox, Constance Gilman, Nicola Turner, Kate Flemming, Lorna K Fraser

**Affiliations:** 1 Department of Oncology and Metabolism, University of Sheffield, Sheffield, UK; 2 Palliative Care Unit, University of Liverpool, Liverpool, UK; 3 The Medical School, University of Sheffield, Sheffield, UK; 4 Sheffield Teaching Hospitals NHS Foundation Trust, Sheffield, UK; 5 School of Health and Related Research, University of Sheffield, Sheffield, UK; 6 St Luke’s Hospice, Sheffield, UK; 7 Academic Unit of Palliative Care, School of Medicine, University of Leeds, Leeds, UK; 8 Department of Health Sciences, The University of York, York, UK; 9 Department of Health Sciences, Martin House Research Centre, University of York, York, UK

**Keywords:** Child, adolescent, palliative care, quality of death, quality of dying, terminal care, tools, review

## Abstract

**Background:**

The circumstances and care provided at the end of a child’s life have a profound impact on family members. Although assessing experiences and outcomes during this time is challenging, healthcare professionals have a responsibility to ensure high quality of care is provided.

**Aim:**

To identify available tools which measure the quality of dying, death and end-of-life care for children and young people; describe the content, and data on validity and reliability of existing tools.

**Design:**

Scoping review was conducted following the Arksey and O’Malley methodological framework.

**Data sources:**

Four electronic databases (MEDLINE, EMBASE, CINAHL and PsycINFO) and grey literature were searched for studies published in English (January 2000–June 2021). A review of reference lists and citation searching was also undertaken. Tools needed to include a focus on the ‘dying’ phase of illness (defined as the last month of life).

**Results:**

From 2078 articles, a total of 18 studies, reporting on 11 tools were identified. All tools were completed by primary caregivers or healthcare professionals as ‘proxy’ assessments; all except one was undertaken after death. Question items about quality of life and preparation for death were found in all tools; items relating to cultural aspects of care, grief and financial costs were less common. Only 6/11 had undergone psychometric testing within a paediatric palliative care setting.

**Conclusions:**

Future research should include ways to adapt, refine and improve existing tools. Assessing their wider application in different clinical and cultural settings and conducting further psychometric assessment represent areas of focus.

**What is already known about the topic?**

- The circumstances and care received at the end of a child’s life can have a profound effect on parents and siblings.
- Measuring experiences and outcomes during this time is challenging but extremely important to ensure high quality of care is provided.

**What this paper adds**

- This is the first scoping review to systematically identify tools assessing the quality of dying, death and end-of-life care for children and young people.
- Gaps were identified in the assessment of salient domains relating to cultural aspects of care, economic costs and grief.
- Only 6 of the 11 tools had conducted specific psychometric testing within a paediatric palliative care setting.

**Implications for practice, theory or policy**

- Rather than developing new tools, future focus should include ways to adapt, refine and improve existing ones.
- Further work is needed to determine whether the existing tools are suitable for use in a wider cultural context.
- The direct views of the dying child and those of the sibling are not captured by existing measures.

## Introduction

Despite marked improvements in health services, medical treatments and public health, over 4600 babies, children and young people aged 0–19 years die each year in high-income countries, such as the United Kingdom (UK).^1^ Globally, the Lancet Commission highlighted that 2.5 million children die each year with ‘serious health related suffering’, with the majority of deaths occuring in low and middle income countries.^2^ Therefore, a large number of parents and other family members worldwide suffer the consequences of a child bereavement. The effects of the death of a child on parental health and wellbeing are well known.^3–6^ However, the circumstances and care received at the end of a child’s life can have a profound effect on parents and siblings in terms of their subsequent relationships, roles, friendships, and ability to carry on with their lives.^7^ The key elements of a ‘good death’^8^ from the perspective of a dying child, the child’s family and the healthcare providers, include: preserving quality of life; preparation for death; specific aspects of care such as continuity, addressing cultural and spiritual concerns; and considering the impact on survivors.^9^

The period of care up to and during the end of a child’s life is extremely important and healthcare professionals have a responsibility to ensure high quality care, including dignity, respect and symptom control, is provided during this time. Defining high quality care at the very end of life is greatly dependent on the preferences and priorities of the patient and their family and their views are central to any efforts to measure quality. Measuring care, outcomes and experiences during end-of-life is challenging but patient reported outcome measures (PROMs) can be used.^10^ Although the patient’s perspective on the quality of end-of-life care should be sought whenever possible, this is not easy, especially with children. Children receiving palliative care may be non-verbal, too young or too unwell to complete self-report tools.^11^ Debate also exists about who is best placed to complete outcome measures for children and young people.^11^ Potential ‘proxy’ assessments can be undertaken by a parent, carer, or professional, but their degree of agreement with child self-report measures is variable. For example, child and parent scores tend to be better correlated for more observable, physical aspects of care and poorer for issues such as emotional problems.^12^

When evaluating outcomes, it is often the case that a range of PROMs are available that could be used for a given purpose (i.e. to assess quality of end-of-life care and death). Reviews and evaluation work are therefore necessary for researchers and clinicians to help map what tools are available and their supporting psychometric evidence. In adults, a number of systematic reviews have identified, appraised and assessed tools used with ‘proxies’ that is bereaved family carers after the death to measure quality of end-of-life care.^13–16^ None have specifically focussed on tools used to assess quality of dying, death and care at the very end of life for children and young people.

Scoping reviews represent a way of mapping broad areas; they provide breadth, as compared to depth, and help identify any research gaps in the literature.^17^ Within this scoping review, we aimed to address the following research question:

What existing tools are available to measure the quality of dying, death, and end-of-life care for children and young people?

An additional sub-question was:

What can we determine about the quality of these tools e.g., comprehensiveness of content, assessment for validity and reliability (as demonstrated by their development process and reported psychometric testing)?

## Methods

### Design

The scoping review was conducted in five stages following the Arksey and O’Malley^17^ framework: identifying the research question; identifying relevant studies; study selection; charting the data; and collating, summarizing and reporting the results. Additionally, we incorporated enhancements to this original framework using the Joanne Briggs Institute guidance (https://jbi.global/scoping-review-network/resources). Reporting was informed by the PRISMA extension for Scoping Reviews (PRISMA-Scr).^18^

### Search strategy

Working in collaboration with a subject librarian (MC), an initial limited search of EMBASE was undertaken to identify relevant target papers. Text words within titles and abstracts and the index terms of these articles were used to generate a full search strategy. The search strategy consisted of four main concepts: ‘quality of death’, ‘tool’, ‘palliative care’ and ‘children and young people’ (Textbox 1). We defined ‘children and young people’ as those less than 25 years of age, to include adolescents as well as younger children.^19^ We did not include studies which focussed solely on neonatal deaths (within first 27 days of life)^1^ as these tend to relate to perinatal factors,^1^ infections and premature birth.^20^ For the purposes of this review, the ‘dying period of their illness’ was regarded as the last month of life, reflecting that advanced, incurable illnesses have different disease trajectories. Where a specific time period was not stated, tools which had specific questions about the quality of dying or death were also included.

The National Institute for Health and Care Excellence (NICE) guidance for end-of-life care for children^21^ was used to inform the chosen search terms under each search concept. Modifications were made, for example, to ensure the search strategy focussed on the ‘dying period’ rather than the broader remit of palliative care. An electronic literature search was conducted on 15th June 2021 with four electronic databases (MEDLINE, CINAHL, EMBASE and PsycINFO) covering the years from January 2000 to June 2021 (Supplemental file 1). This time period reflects more recent changes within paediatric palliative care (e.g. formation of the Association of Paediatric Palliative Medicine within the UK (https://www.appm.org.uk/)). Specified inclusion and exclusion criteria (Textbox 1) were used to identify studies.

**Textbox 1.** Inclusion and exclusion criteria.

Inclusion criteria
• Focus on tools used to assess quality of death, dying or quality of care at the end of life • Participants are children or young people identified as dying OR parents/family members/ carers/healthcare professionals caring for dying children or young people OR recently bereaved parents/family members • Published studies of any research design
Exclusion criteria
• Focus only on neonates or individuals >25 years old • Focus on tools, used with children/young people with a life-limiting illness, BUT have not been used to assess the dying period of their illness (defined for the purpose of this review as ‘last month of life’) • Articles such as case studies, case series, books, editorials, commentary or opinion pieces or conference abstracts • In language other than English

Titles and abstracts were initially screened by teams of two independent reviewers. A full text review of all potentially eligible studies was conducted independently the same teams; any areas of uncertainty were resolved by discussion with the lead author. Review articles were not included, but reference lists were screened to identify any additional papers. A citation search of all selected articles was completed, and reference lists of all included papers were screened for potentially relevant studies. Grey literature was also searched using the search terms *‘palliative care’ AND (child OR children) AND* (*questionnaire* OR *survey*) AND ‘*quality of death*’. These included Internet searches of Google, World Health Organization Europe, NICE and Royal College of Nursing. Specific organization websites were reviewed for information on potential tools.

### Data extraction

Data was extracted using a specially designed proforma (piloted prior to use) by one member of the research team and verified by the lead author. Data was mapped out, using selected principles developed by the Scientific Advisory Committee of the Medical Outcomes Trust to assess quality-of-life instruments,^22^ namely: conceptual model and reported psychometric testing (validity and reliability). The content of each tool was mapped to the seven key dimensions of a ‘good death’ (from research which incorporated the perspectives of the dying child, the child’s family and healthcare providers).^9^ These considerations were supplemented by information on the study objective, tool purpose and description, assessment period, setting, population, participants and key study findings.

### Collating and summarizing data

Charted data were then tabulated into the following categories, reflecting the predominant use of the tools:

1. Healthcare professional: tool used solely with healthcare professionals
2. Cancer: tool used solely within a cancer population
3. Cancer and non-cancer: tool used in populations with more than one disease groups (both malignant and non-malignant illnesses)
4. Life-limiting cardiac disease: tool used solely within an advanced cardiac disease population.

Comparisons were made between the extracted results focussing on development and use, content, participants and psychometric testing. This method highlighted dominant areas and allowed gaps to be identified. In keeping with the accepted remit of scoping review guidance, specific quality appraisal (e.g. of the methodology or psychometric properties), was not conducted. Rather, where documented within the manuscripts, these details were directly extracted. Where specific details were missing about tool content, the corresponding author of the relevant study was contacted and invited to provide further information.

## Results

### Range of studies

**Figure 1.**
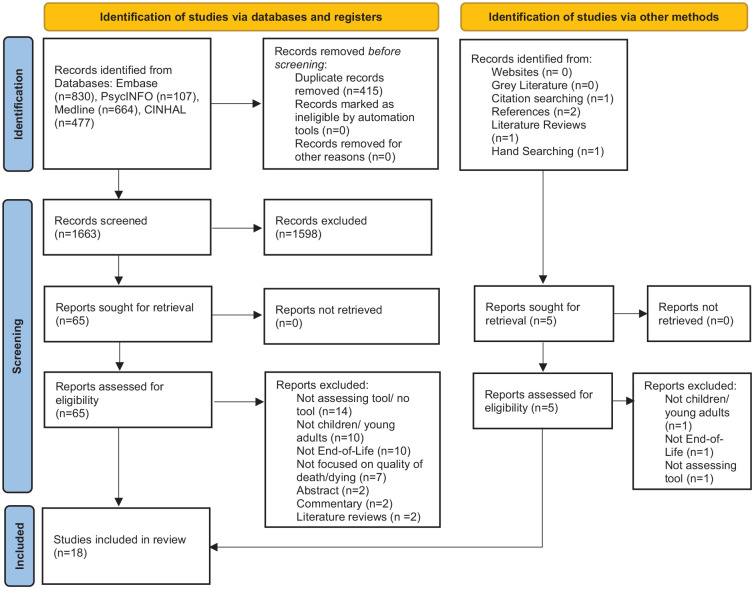
PRISMA flow diagram for the scoping review process.

The initial search identified 2078 articles across all databases. Removal of duplicates resulted in 1663 papers, 65 of which were retrieved for full text review. A further 49 papers were excluded on reviewing full papers (Figure 1). Two additional articles were identified through reference lists and citation searches. A total of 18 papers^23–40^ were included in the review, reporting on 11 tools. One study reported on the use of two different tools,^27^ whereas all other studies used a single tool.

The 18 studies were conducted in seven countries: USA (*n* = 9)^25,27–29,35,36,38–40^; Japan (*n* = 2)^22,24^; Switzerland (*n* = 2)^32,33^; Germany (*n* = 2)^30,31^; Canada (*n* = 1)^37^; South Korea (*n* = 1)^26^ and Spain (*n* = 1).^34^ Twelve of the studies involved children’s hospitals: paediatric oncology ± haematology departments (*n* = 5)^23,24,26,28,31^; paediatric cardiology centres (*n* = 2)^39,40^; paediatric intensive care units (*n* = 2)^25,38^; medical centres/hospitals (*n* = 2)^27,30^; or mixed hospital environments (*n* = 1).^34^ The remaining six studies involved hospital and community settings (e.g. home care).^29,32,33,35–37^ The primary objective of the studies varied, with the two main aims being to develop and test a tool^24,25,32,35,37^ or to explore perspectives of parents and/or healthcare professionals about the quality of dying and end-of-life care experiences.^23,26–31,33,34,36,38,39^ Study participants comprised parents only (*n* = 10)^26,28–34,39,40^; healthcare professionals only (*n* = 3)^23–25^; parents and partners (*n* = 1)^27^; parents and guardians (*n* = 1)^38^ and parents and healthcare professionals (*n* = 3).^35–37,40^ In total, there were 1859 participants involved in the development, validation or use of tools, representing 1048 children and young people. For studies involving family caregivers, participants tended to be female (range 56%–100%) and, when specified, from a white ethnic background (range 72.9%–100%).

### Range of tools

The 11 tools were sub-categorized into the defined groups: sole use by healthcare professional tools (*n* = 2) (Table 1); tools used within a cancer population (*n* = 4) (Table 2); tools used with both cancer and non-cancer populations (*n* = 4) (Table 3) and tools used solely within a life-limiting cardiac disease population (*n* = 1) (Table 4). The content of each tool was mapped to the key dimensions of a ‘good death’ (Table 5).

**Table 1.** Studies detailing the development, validation and initial use of healthcare professional tools assessing quality of dying, death and end-of-life care for children/young adults.

	Study objective	Purpose of tool and underlying concepts	Description of tool and specified assessment period	Details of tool development	Setting	Population	Participants	Reported psychometric testing	Key findings including any quality of dying, death, EOL evaluations
*Good death Inventory – Paediatrics (GDI-P)*									
Nagoya et al.^23^	To identify and describe important items and concepts related to QoL for paediatric cancer patients’ EOL in Japan	To evaluate QoL of paediatric cancer patient’s EOL Care Four dimensions-physical, psychological, social and spiritual	Used after-death Questionnaire – survey initial 55 items reduced to 35 items Response options on a 5-point Likert-type scale from ‘very important’ to ‘not important’ Time phase = ‘time before death when the physician estimated that the child had no realistic chance for cure’; items include *‘dying in presence of family’*	Items developed from previous qualitative research (seven bereaved families, seven paediatric oncologists and 13 nurses – published in Japanese)	Japan Nationwide survey of 75 paediatric oncology treatment facilities	Directors of 46 paediatric oncology institutes and 49 nursing institutes who had at least 1× EOL care experience	157/253 oncology directors (RR 62.1%); 48 (31%) female; mean age 40.53 year (SD 8.75); ethnicity N/S 270/646 nursing directors (RR 41.8%); 254 (94.8%) female; mean age 34.35 year (SD 8.79); ethnicity N/S	Face validity assessed by four nurses; 35 items rated ‘very important/important’ by >80% respondents EFA identified 12 QoL domains: Playing and learning; Fulfilling wishes; Spending time with family; Receiving relief from physical and psychological suffering; Making wonderful memories; Having a good relationship with the staff; Having a peaceful death in the presence of family; Spending time with a minimum of medical treatment; Living one’s life as usual; Spending time in a calm hospital environment; Being oneself; Having a close family	Identified 35 common, important QoL items for assessing EOL care in paediatric cancer patients
Nagoya et al.^23^	To develop and test a proxy rating scale assessing QoL of paediatric cancer patients receiving EOL Care To develop a shortened version of GDI-P	To assess QoL of paediatric patients receiving EOL care, as perceived by nursing staff eight main factors: A peaceful death in the presence of family; Relief from physical and psychological suffering; Playing and learning; Making wonderful memories and fulfilling wishes; Living a normal life; Good relationships with medical staff; Spending time with the family; Minimum medical treatment	Used after-death Questionnaire – survey GDI-P: eight factors with 22 items Response options on a 5-point Likert-type scale from ‘strongly agree’ to ‘strongly disagree’ Higher scores = greater degree of achievement for that item Time phase = ‘time before death when physicians estimated the child had no realistic chance of being cured’	Developed from previous qualitative and quantitative work (see above) Tested for face validity (four nurses) and pilot study (*n* = 7, six nurses and one physician) at single study centre Short version GDI-P: eight items (one from each factor)	Japan 60 paediatric facilities including hospitals for childhood cancer	Paediatric nurses working in EOL care Cared for child (⩽20 year) who died from cancer (Oct 2012–Oct 2015) Child’s family been told child was in EOL phase Asked for two nurses’ perceptions per child	85/112 completed QA (RR 76%) 32 pairs (64 QA) where two nurses evaluated single child; 21 single assessments Mean age 31.9 year (SD 7.5); 81 (95%) female; ethnicity N/S Representing 53 children; mean age 8.5 year (SD 4.9); most died in general hospital ward (84%); also deaths in ICU, home and ‘unknown’ 47 retest QA returned	Good internal consistency (Cronbach’s α 0.71–0.87 for each factor; overall scale 0.88) Construct validity assessed by convergent and discriminant validity testing Low GFI < 0.90 – potentially due to small sample size ICCs for test-retest moderate-good (0.61–0.94) Short version GDI-P: correlations between item-overall scores ranged from 0.82 to 0.91; Cronbach’s α = 0.67 for all eight items	GDI-P usable as a proxy outcome measure assessing EOL phase of illness for paediatric cancer patients
*Paediatric Intensive Care Unit - Quality of Dying and Death 20 (PICU-QODD-20)*									
Sellers et al.^25^	To develop and assess reliability and validity of a clinician measure of the quality of dying and death in the paediatric intensive care setting	To assess ‘the degree to which the hopes and priorities of the patient and/or the family for the process of dying and the moment of death are respected and met’ Key themes within final items: Communication issues; Privacy and PICU environment issues; Decisions to withdraw life support; Pain and symptom management; Emotional needs/support of family; Physical and instrumental needs of family; Spirituality and religion/cultural issues; Continuity/coordination of care; Fulfilling the parental role; Grief and bereavement	Used after-death Questionnaire – survey Final version has 20 items; each has 11-point scale (0 = ‘as terrible’ to 10 = ‘as good as it could be, under the circumstances’) Standardized score out of 100; higher scores = more positive experience Time phase = last 3 days of life	Adapted from adult version of QODD Developed using focus groups with PICU clinicians; qualitative interviews with parents of children who died in a PICU and cognitive interviews; systematic literature review	USA PICU’s from two large children’s hospitals	Five types of HCP for each child’s death: ‘bedside’ nurse; child’s primary nurse; child’s intensivist; most involved critical care fellow and other clinician (psychosocial staff) To children who died in a PICU over 12-month period from 2008 (multiple different causes of death)	300/551 completed QA (RR 54%) Percentage of distributed QA completed by: ‘bedside’ nurse 55%, primary nurse 50%, intensivist 57%, fellow 47%, other clinician 61%, 33%–95% female; 5%–27% non Caucasian; age N/S Representing 94 children; mean age 7.3 year (SD 7.2); range 0–24 years; ‘*just under half were female’*; ethnicity not consistently recorded	Good internal consistency (Cronbach’s α = 0.891–0.959) Construct validity assessed by comparison with other measures: total PICU-QODD-20 score significantly related to single-item ‘quality of EOL care’ and ‘Meeting Family Needs’ scale (*r* = 0.333–0.797) Hypothesized that ‘family barriers’ (e.g. anger, unrealistic expectations) associated with poorer experiences of dying and death; PICU-QODD-20 negatively associated with ⩾2/8 potential barriers for all clinicians except bedside nurses	Findings provide initial support that PICU-QODD-20 is valid and reliable outcome of the quality of dying and death in the PICU setting

EFA: exploratory factor analysis; EOL: end-of-life; GFI: goodness of fit index; HCP: healthcare professional; ICC: intraclass correlation; N/S: not stated; PICU: paediatric intensive care unit; QA: questionnaire; QODD: Quality Of Dying and Death; QoL: quality of life; RR: response rate; SD: standard deviation; USA: United States of America.

**Table 2.** Studies detailing the development, validation and initial use of tools assessing quality of dying, death and end-of-life care for children/young adults within a cancer population.

	Study objective	Purpose of tool and underlying concepts	Description of tool and specified assessment period	Details of tool development	Setting	Population	Participants	Reported psychometric testing	Key findings including any quality of dying, death, EOL evaluations
*Good Death Inventory (GDI)*									
Kim and Park^26^	To assess essential domains for a ‘good death’, using the GDI, as perceived by parents whose children have cancer To examine characteristics associated with perceptions of a good death	To evaluate perceptions regarding EOL care from the perspective of bereaved family members 10 core domains: Physical and psychological comfort; Dying in a favourite place; Maintaining hope and pleasure; Good relationships with medical staff; Not being a burden to others; Good relationships with family; Independence; Environmental comfort; Being respected as an individual; Life completion	Used after-death (although this developmental work was conducted prospectively before death) Questionnaire – survey 18 domains (10 core, 8 optional); each domain has three items Revised original GDI tool so each participant rated the importance of each item using 7-point Likert scale (1 = absolutely unnecessary to 7 = absolutely necessary) Total GDI score = 18–126 (higher scores = good death) Time phase = not specified but domains include focus on death/dying	Previous translation into Korean and validated within adult population	South Korea Outpatient clinic of Paediatric Haematology and Oncology department; single university hospital	Parents to children (aged 7–18 years) who had undergone any stage of cancer treatment	109/120 data analysed (11 had incomplete data) 93 (85.3%) female (85.3%); age and ethnicity N/SRepresenting 109 children; mean age 9.65 year (SD 5.88); 60 (55%) male; ethnicity N/S	Face validity of revised GDI evaluated by three parents; parents within current study also ‘evaluated the validity of revised GDI’ Good internal consistency (Cronbach’s α −0.87)	Mean total GDI score was 107.47 (SD 6.02) Most important domains (had highest scores) were ‘maintaining hope and pleasure’ and ‘being respected as an individual’ Perception of good death (highest GDI scores) associated with following factors: children had discussed EOL plans with parents; agreement between children and parents to establish a living will
*Family Satisfaction with End-of-life Care (FAMCARE)*									
Currie et al.^27^	To understand bereaved caregiver perspectives’ (to adolescents/young adults (AYA)) about EOL care and quality of EOL communication	To measure family satisfaction with advanced cancer care four domains: Family satisfaction with cancer care; Satisfaction with communication with HCP; Availability of clinicians; Pain and symptom management	Used after-death Questionnaire – survey 20 items; 5-point nominal scale from ‘very dissatisfied’ to ‘very satisfied’ Time phase = not specified (but used concurrently with tool below)	Established tool previously used and validated with bereaved families for adult deaths	USA three academic medical centres with Palliative Care Research Cooperative sites within three different states	Bereaved primary caregivers To deceased oncology AYA (aged 15–39); died 2013–2016	35/260 bereaved caregivers completed QA(13.5% RR) 25 (71%) female; 30 (86%) white; age N/S; 15 (44%) spouse/partner; 17 (50%) parent Representing 35 AYA; 11 (31%) < 25 year; 15 (43%) female; 28 (80%) white	Not specifically undertaken within this study	Most caregivers satisfied with EOL care; six (17%) caregivers dissatisfied with information about prognosis, answers from HCP and availability of doctors
*The Toolkit After-Death Bereaved Family Member Interview (TIME)*									
Currie et al.^27^Same study as above	As above	To measure quality of EOL care Conceptual model of patient-focussed, family centred medical care Toolkit After-Death Bereaved Family Member Interview, previously used with bereaved families for adult deaths	Used after-death Questionnaire – survey 64 items; mix of dichotomous and scaled responses (further details not provided in study) Time phase = not specified but question items include focus on death/dying for example ‘*was information given about what to expect about dying?’*	Established tool previously used and validated with bereaved families for adult deaths	As above	As above	As above	Not specifically undertaken within this study	Unmet needs about what to expect at time of death (*n* = 17, 50%), the dying process (*n* = 15, 45%) and spiritual/ religious needs (*n* = 13, 38%) Lowest quality of EOL care scores related to communication and emotional support
*Questionnaire initially developed by Wolfe et al; subsequently called ‘Survey about Caring for Children with Cancer’ (SCCC)*									
Wolfe et al.^28^	To determine patterns of care, symptoms in last month of life, effectiveness of their treatment and factors associated with suffering from pain at EOL for children who die of cancer	Purpose of tool linked to study objectives: To determine patterns of care, symptoms in last month of life, effectiveness of their treatment and factors associated with suffering from pain at EOL for children who die of cancer	Used after death Questionnaire – face-to-face or telephone interview 211 items assessing symptoms; degree to which child ‘appeared to suffer’ (5-point Likert scale); effectiveness of treatment; anxiety, fear, mood; quality of life (determined by ‘degree to which he/she had fun’); degree of physician involvement in EOL care; quality of care and communication; involvement of home care staff; decisions and ‘peacefulness of the child’s death’ Time phase = last month of life	Question items developed from literature, parent and HCP focus groups, and existing validated surveys	USA Single institution (children’s hospital and cancer institute)	Bereaved parents To children who had died from cancer (1990–1997)	103/165 bereaved parents completed interviews (62% RR) Mean 43 year (SD = 7.7); 86% female; 91% white Representing 103 children; mean age 10.8 (SD 6.7); 46 (45%) female; ethnicity N/S	Not specifically undertaken within this study Instrument was assessed for content, wording, burden on respondents, cognitive validity, and willingness to participate; found to be ‘satisfactory’	89% reported their child experienced ‘a lot’ or ‘a great deal of suffering’ from ⩾ 1 symptom (most common were fatigue, pain, dyspnoea, poor appetite) 70% described their child’s death as ‘very peaceful’ ‘Suffering’ from pain more likely reported when physician not actively involved in providing EOL care (OR 2.6)
Friedrichsdorf et al.^29^	To compare EOL pain and symptom management in children with advanced cancer who received care from a paediatric oncology service (Oncology) with those who also received concurrent PPC home care services (PPC/Oncology)	As above – to evaluate EOL care domains Specific domains assessed in this study: Symptoms and their treatment; Quality of life	Used after-death Questionnaire – survey Contains 211 items; prevalence of symptoms, ‘suffering’ from these, management; decision-making at the EOL; quality of life Time phase = parents recalled aspects of their child’s QoL during the last month of their life	As above	USA two children’s hospitals within single state (including those who had in-patient, out-patient or home care/home hospice services)	Bereaved parents to children (aged 0–17 years) who died of cancer (2002–2008)	60/166 surveys obtained (RR 37%); 50% PPC/Oncology Mean age 43.6 year (SD 7.7); 48 female (81%); 56 white (93%) Representing 60 children; mean age 10.1 year (SD 6.3); 27 (45%) female; ethnicity N/S	Not specifically undertaken within this study	PPC/Oncology group more likely to have constipation (*p* = 0.01) and perceived to ‘suffer’ from energy loss/fatigue (*p* = 0.007) PPC/Oncology group more likely to have ‘fun’ (70% vs 45%, *p* = 0.03), to experience ‘an event that added meaning’ to life (89% vs 63%, *p* = 0.02), and to die at home (93% vs 20%, *p* < 0.0001)
Hechler et al.^30^	To investigate bereaved parents’ perspectives on: symptoms and QoL; characteristics of child’s death; anticipation of child’s death and care delivery; EOL decisions; impact of death on parents	Used German version of questionnaire developed by Wolfe (see above)	As above Assessing symptoms, QoL, quality of care, burdens after child’s death Time phase = time span when parents aware there was ‘no realistic chance of their child being cured of cancer’ (parents assessed EOL period as average 9 week prior to death)	Translated into German; minor modifications; pilot with children’s oncologists, nurses, psychologists and interviews with 10 bereaved parents	Germany 6/19 children’s hospitals within single state	Bereaved parents to children who had died from cancer (1999–2000)	48/136 bereaved families participated (35% RR); 40 interviews with single parent, eight with both parents; demographics N/S Representing 48 children; 17 (35%) female; mean age 8 year (SD 4.9), range 1–20; ethnicity N/S	Not specifically undertaken within this study	Fatigue (*n* = 40, 91%) and pain (*n* = 35, 83%) most common symptoms; dyspnoea and anxiety caused most ‘suffering’ and were less adequately treated 48% children died at home; in hindsight, 88% participants would have chosen home as most appropriate place; 88% rated quality of care for home care team as ‘good’/‘very good’ seven (15%) weren’t contacted by team following death
Von Lützau et al.^31^	To investigate bereaved parents’ perspectives on: symptoms and QoL at EOL; perspectives about impending death; palliative home care; quality of care EOL decision-making; characteristics of death	Used German version of questionnaire developed by Wolfe (see above)	As above Assessing symptoms, QoL, quality of care, burdens after child’s death Time phase = time span when parents aware there was ‘no realistic chance of their child being cured of cancer’ (parents assessed EOL period as average 8.5 week prior to death)	As above	Germany 16 specialized paediatric oncology departments (hospital setting) within single state	Bereaved parents to children who died from cancer (2005–2006)	48/128 bereaved families participated (RR 38.3%); 37 interviews with single parent, 11 with both parents; 35 female (72.9%); age and ethnicity N/S Representing 48 children; 11 (22.9%) female; mean 9.93 year (SD 7.3); ethnicity N/S	Not specifically undertaken within this study	Results suggested some improvement in EOL care c.f. above study Fatigue (*n* = 44, 91.7%) and pain (*n* = 40, 83.3%) most common symptoms; 65% symptoms adequately treated; 84% with ‘severe’ pain treated successfully’ 43.8% children had psychological support 24 (50%) died at home; in hindsight, majority (72.9%) of parents would not have changed preference for place of death

AYA: adolescents and young adults; EOL: end-of-life; HCP: healthcare professional; N/S: not stated; OR: odds ratio; PPC: paediatric palliative care; QA: questionnaire; QoL: quality of life; RR: response rate; SD: standard deviation; USA: United States of America.

**Table 3.** Studies detailing the development, validation and use of tools assessing quality of dying, death, and end-of-life care for children/young adults within a mixed cancer and non-cancer population.

	Study objective	Purpose of tool and underlying concepts	Description of tool and specified assessment period	Details of tool development	Setting	Population	Participants	Reported psychometric testing	Key findings including any quality of dying, death, EOL evaluations
*Parental PELICAN Questionnaire (PaPEQu)*									
Zimmermann et al.^32^	To develop and test the Parental PELICAN Questionnaire (PaPEQu)	To assess parental experiences and needs during EOL care of their child Items generated from six quality domains grounded in framework of the ‘Initiative for Paediatric Palliative Care’ Holistic care of the child; Support of the family unit; Involvement of child and family in communication, decision-making and care planning; Relief of pain and other symptoms; Continuity of care; Grief and bereavement support	Used after-death Questionnaire – survey Separate questionnaires for four different diagnostic groups; items organized into scales about parental experiences and indexes for parental needs Experience-related items, 7-point adjective response options or 5-point Likert scale response options with varying end-point anchors for example, ‘never-always’, ‘not clear at all-very clear’ Needs-related items, 7-point adjectival response options with end-point anchors ‘not important at all-very important’ Overall satisfaction with each of the six domains (7-point scale) Additional: to list three positive and negative EOL experiences; indicate areas of life negatively influence by child’s death; rate current QoL (10-point VAS) Time phase = not specified but used for care within last 4 week of life in study below	Development (four phases): 1. Item generation; 2. Validity testing with HCP expert panel (including I-CVI calculations) and cognitive interviews with bereaved mothers (*n* = 4); 3. Translation (from German into French/Italian); 4. Pilot survey	Switzerland Pilot: children’s hospitals (*n* = 3) /paediatric hospital dept (*n* = 1)/paediatric medical centre (*n* = 1) Main study: children’s hospitals/paediatric units (*n* = 17), long-term institutions (*n* = 2) and community care services/practices (*n* = 6)	Pilot: bereaved parents (*n* = 36) To children who had died due to cardiac, neurological or oncological illness or during first 4 weeks of life Main study: bereaved parents To child who died (same conditions as above) during 2011–2012	Pilot: 36 families invited; 31 QA sent (mother and father versions) to 20 families; 24 completed QA (77% RR) Main study: 200/224 completed QA (89% RR) representing 124 families; 112 (56%) mothers; 88 (44%) fathers; age N/S No ethnicity data reported, but language = 162 German (81%), 29 French (14.5%), 9 Italian (4.5%) Representing 124 children; median age 3.3 year (range 0.1–17.4); gender and ethnicity N/S	Development phase: average CVI > 0.78; feedback used to reduce/revise items Main study: EFA showed one factor for each scale supporting uni-dimensionality Correlations between scale mean and satisfaction score statistically significant (0.37–0.63)	Psychometric testing of six quality domains showed uni-dimensionality and internal consistency of each domain
Zimmermann et al.^33^	To describe parental experiences and explore differences in perspectives in relation to underlying medical condition causing death (cardiac, neurological or oncological condition or during the neonatal period)	As above	As above Experience related items range 44–48 items (depending on diagnostic group version); 34 needs-related items; and 13 socio-demographic items Total item count of the PaPEQu range 91–95 items. Time phase = last 4 weeks of life	As above	Switzerland as above (main study): Paediatric hospital (*n* = 17) and community care settings (*n* = 8)	As above (main study): Bereaved parents to children who died due to cardiac, neurological or oncological condition or during the neonatal period (2011 or 2012)	As above (main study) 200/224 completed QA (89% RR); 112 (56%) mothers; 88 (44%) fathers; mean age 40 year (SD = 6.48); Swiss residents 87%, migrant families 13%; representing deaths due to cardiac (26, 13%), neurological (48, 24%), oncological (45, 22%) illness or during neonatal period (81, 42%) Representing 124 children median age 3.3 year (range 0.1–17.4); gender/ethnicity N/S	As above	Experience scores highest for ‘relief of pain and other symptoms’ (mean 4.99, SD − 1.05); lowest for ‘continuity and coordination of care’ (mean 4.29, SD = 1.37) Highest perceptions for cancer EOL care (mean 4.80, SD = 0.51); lowest for neurological conditions (mean 4.51, SD = 0.44)
Plaza Fornieles et al.^34^	To assess effectiveness of the PPC team To assess whether involvement of the PPC team improved EOL care based on experiences and parents’ level of satisfaction with care	As above	As above	Translated Italian version of the PaPEQu into Spanish using international guidelines	Spain Department of Paediatrics in single university hospital three groups: 1. PPC group (managed by PPC team); 2. Non-PPC group (managed by paediatricians not specialized in PPC); 3. Neonatal group (managed by neonatal intensive care unit team)	Bereaved parents to children who died (June 2014–June 2017) from life-threatening/life-limiting disease	Two copies of QA sent to 55 families (one for father; one for mother) 46/108 completed QA (42.6% RR) (two single parent households) 26 (56.5%) mothers, mean age 32.96 year (SD 8.7); 18 (36.7%) fathers, mean age 36.71 year (SD 5.7); 41 Spanish (89.1%); five ‘immigrants’ (10.9%) – Moroccan, Honduran, Ecuadorian, Ukrainian Representing 28 children mean age 42.21 month, 16 female (57.1%); deaths due to cardiac (1, 3.6%), neurological (6, 21.4%), oncological (9, 32.1%) illness or during neonatal period (12, 42.9%); ethnicity N/S	As above	PPC group had highest scores (experiences and satisfaction) for family support, communication, shared decision-making, bereavement support (*p* < 0.05) Neonatal group had least positive experiences Greater proportion of PPC group involved in decisions about CPR, withdrawal of treatment
*EXPERIENCE @Home Measure*									
Boyden et al.^35^	To develop and conduct preliminary evaluation of a family-reported measure of experiences with paediatric palliative and hospice care at home – PPHC@Home	To assess family-reported experiences of palliative and hospice care for children and caregivers at home National Consensus Project’s Clinical Guidelines for Quality Palliative Care used as framework Initial 20 domains reduced to 16 final domains: Access to care; Caregiver support at EOL; Communication at EOL; Communication between family and care team; Coordination of care; Continuity of care; Cultural aspects of care; Ethical and legal aspects of care; Knowledge and skills of care team providers; Physical aspects of care; Practical aspects of care; Psychological and emotional aspects of care; Extended social network; Relationship between family and care team; Social aspects of care; Spiritual and religious aspects of care	Used before death – retrospectively assess care provided during previous week (although in this development work also assessed with bereaved parents) Questionnaire – survey Initial pool of 70 items – final measure had 22 items; 5-point Likert scale from ‘strongly disagree’ to ‘strongly agree’ Time phase = not specified but question items include *‘what my child’s last weeks of life’* may be like	Phase 1: Item identification and development (using guidelines, peer-reviewed literature, existing instruments, key stakeholder feedback). Phase 2: Initial prioritization and reduction of items by HCP using discrete choice experiments (DCE). Phase 3: Final prioritization and reduction of items by parents using DCE. Phase 4: Cognitive interviewing with parents	USA Home-care setting Phase 2: Hospital, community, academic institutions (USA and Canada). Phase 3 and 4: Children’s hospital and virtual community of parents	Phase 2: HCP/parent advocates. Phase 3 and 4: Parents and bereaved parents To children (<25 year) with/died from ‘serious illness’ – either receiving/ previously received PPHC@ Home	Phase 2: 37 HCP/parent advocates; 31 (91.2% female and white); mean age 48.4 year (SD 9.7). Phase 3: 47 parents; mean age 42.6 year (SD 8.5); 44 (93.6%) mothers; 42 (89.4%) white (further details in study below). Phase 4: 11 parents (subgroup of phase 3); mean age 43.8 years, (SD 6.5); 10 (90.9%) mothers; 11 (100%) white Representing children mean age 9 year (SD 6.4); 3 (27.3%) female; 8 (72.7%) white; range of diagnoses (neurological, cardiac, oncological, genetic)	Not specifically undertaken within this study – identified as next step	Multi-method, multi-stakeholder approach used for instrument development First tool specifically measuring family-reported experiences of palliative and hospice care at home
Boyden et al.^36^	To explore how parents’ rate and prioritize different domains of paediatric palliative and hospice care at home - PPHC@Home (detailing Phase 3 of above study)	As above 20 specific domains	As above	As above – Phase 3 (DCE with parents/bereaved parents)	As above	As above	As above – Phase 3 47 parents; 14 (29.8%) were bereaved; 33 (70.2%) were currently caring for their child at home; mean age 42.6 year (SD 8.5); 44 (93.6%) mothers; 42 (89.4%) white Representing 45 children; 21 (46.7%) female; >50% aged 10–25 years; 37 (82.2%) white; most common diagnoses (could have >1): neuromuscular, neurologic, or mitochondrial (51.1%), genetic/congenital (48.9%), cardiovascular (22.2%), metabolic (22.2%)	As above	Overall, highest-rated domains were: Physical aspects of care: Symptom management; Psychological/emotional aspects of care for the child; Care coordination Lowest-rated domains were: Spiritual and religious aspects of care; Cultural aspects of care (but participants were mainly white, non-Hispanic, and Christian)
*Quality of Children’s End-of-Life Care Instrument*									
Widger et al.^37^	To develop and test an instrument measuring quality of EOL care, from the perspective of bereaved mothers	To assess quality of children’s EOL care Instrument designed to measure structure, process, or outcome (in keeping with Donabedian’s model of quality health care) 10 final domains: Connect with families; Involve parents; Share information with parents; Share information among HCP; Support the child; Support siblings; Support Parents; Structures of care; Provide care at death; Provide bereavement follow-up	Used after death Questionnaire – survey Revised instrument had 95 items on structures, processes, outcomes; six subscales Most items have five adjectival response options (‘never’ to ‘always’) or are satisfaction ratings; some dichotomous response options Time phase = not specified but includes domains focussing on care provided at death (whether ‘*peaceful death’*)	Phase 1: Literature review – identified indicators of high-quality EOL care. Phase 2: Focus groups – bereaved parents asked about important domains for EOL care. Phase 3: Item development and refinement – HCP to assess content validity and cognitive interviews with bereaved parents. Phase 4: Psychometric testing	Canada Phase 2 and 3: death occurred in hospital or home setting. Phase 4: 10 children’s hospitals and hospices	Phase 2: Bereaved parents. Phase 3: HCP with expertise in paediatric EOL care and bereaved parents. Phase 4: Bereaved mothers To children (<19 years old) who died in a hospice/hospital (2006–2009)	Phase 2: 10 bereaved parents; mean age 44.5 year; 90% Caucasian Representing 10 children (mean age 5 year); 7 female; 4 = cancer, 5 = congenital illness, 1 = neuromuscular condition. Phase 3: 7 HCP were physicians (*n* = 2), advanced practice nurses (*n* = 4), and social worker (*n* = 1); 6 bereaved parents from phase 2. Phase 4: 128/657 bereaved mothers completed instrument (18% RR); further 31 for test-retest assessment; mean age 36.5 year (SD 8.3); ethnicity N/S Representing 128 children, mean age 4.1 year; 66 (51.6% female; ethnicity N/S; most common 1^0^ diagnosis = congenital malformations (23.4%) and neoplasms (16.4%)	Phases 1–3 supported face and content validity. Phase 3: CVI scores for individual items (0.67–1.0) and overall = 0.84 (items scoring <0.8 were revised). Phase 4: EFA only possible for 6/10 subscales (due to missing data, ‘not applicable’ responses); good test-retest reliability (ICC 0.81–0.9) and good internal consistency (Cronbach’s alpha 0.76–0.96) Remaining 4/10 subscales had good content validity	Initial evidence for reliability and validity of six subscales and content validity for four additional subscales
*PICU-QODD*									
Yorke^38^	To explore parents’ experiences of a child’s death in PICU To explore ideas about how to improve experiences	To allow parents to evaluate their and their child’s experience Published ‘Framework for a Good Death’ guided overall research	Used after death Questionnaire – completed PICU-QODD and conducted face-to-face qualitative interview 25 items – each has a initial question with response options on a 5-point scale (‘none of the time’ to ‘all of the time’); then item asking to ‘rate this aspect of your child’s dying experience’ on an 11-point scale (0 = ‘terrible’ to 10 = ‘almost perfect’) Time phase = not specified but focus on care up to and including death	Established tool, QODD, previously used and validated with bereaved families for adult deaths Modified original version to form PICU-QODD - reviewed by PICU nurses (*n* = 3), bereaved parent (*n* = 1) and compared with aspects of care from ‘Framework for a Good Death’	USA Single PICU in an academic children’s hospital	Bereaved parents/guardians to children who died in PICU (2004–2005)	23/80 parents/grandparents participated (28.8% RR); age range 27–63 years; gender/ethnicity N/S Representing 14 children; age range newborn to 20 year; cancer *n* = 4, congenital heart disease *n* = 5; other causes *n* = 5; gender/ethnicity N/S	Internal reliability assessed with Cronbach’s α 0.929 (but small sample size and missing values)	Majority of aspects of care rated highly in PICU-QODD; range of scores 4–10/10; mean score 7.25 (SD 2.11) Item with lowest rating was whether child was able to be fed or feed him/herself Qualitative interview findings suggest parents want more direct communication, to remain present and involved in care and support after the death

CVI: content validation index; DCE: discrete choice experiment; EFA: exploratory factor analysis; EOL: end-of-life; HCP: healthcare professional; ICC: intraclass correlation; N/S: not stated; PPC: paediatric palliative care; PICU: paediatric intensive care unit; PPHC@HOME: paediatric palliative and hospice care at home; QA: questionnaire; QODD: Quality Of Dying and Death; QoL: quality of life; RR: response rate; SD: standard deviation; VAS: visual analogue scale; USA: United States of America.

**Table 4.** Studies detailing the development, validation and use of tools to assess quality of dying, death, and end-of-life care for children / young adults within a life-limiting cardiac population.

	Study objective	Purpose of tool and underlying concepts	Description of tool and specified assessment period	Details of tool development	Setting	Population	Participants	Reported psychometric testing	Key findings including any quality of dying, death, EOL evaluations
*Survey for Caring for Children with Advanced Heart Disease (SCCHD)*									
Blume et al.^39^	To describe bereaved parents’ perspectives whose children died from Advanced Heart Disease (AHD)	Describe parental perspectives of EOL care 10 different domains; four main domains reported within this study: Symptom control; Quality of life; Communication with care team; Use of treatment-directed technologies at EOL	Used after death Questionnaire – survey 110 questions across 10 different domains; items have Likert-style and nominal response options Time phase = not specified but survey includes items focussing on last month of life	Adapted from another questionnaire (developed by Wolfe J et al, 2000 – see Table 2) Items selected based on literature review and adapted to cardiac ICU setting; used items from previously validated questionnaires, where possible Pilot: feedback from 4× parents of deceased children (2× AHD, 2× cancer)	USA Two large paediatric cardiology centres (hospitals) in single city	Bereaved parents To children (<21 years) who died from any type of heart disease (Jan 2007–Dec 2009)	50/128 bereaved parents completed QA (39% RR); 47 (95%) female; median age 37.6 year; 47 non-Hispanic white (94%) Representing 50 children; median age 6 month (range 3.6 days–20.4 years); gender and ethnicity N/S	Not specifically undertaken within this study	47% perceived child ‘suffered’ ‘a great deal/a lot/somewhat’ during EOL Parents to children <2 years perceived breathing and feeding difficulties and fatigue to cause most ‘suffering’ c.f. fatigue and sleeping difficulties in older children 71% reported QoL in last month of life as ‘poor’ or ‘fair’; 84% reported quality of care ‘good’ or ‘excellent’ 14 (40%) realized <= 1 day prior to death that death was imminent; nine (18%) never realized until time of death31 (70%) agreed that their child had experienced a ‘good death’
Balkin et al 2015^40^	To describe and compare primary cardiologists and bereaved parents’ perspectives about care for children who died of AHD	Sub study of original cohort study (see above) Describe parental and physician perspectives of EOL care	Used after death Questionnaire – survey SCCHD: 110 questions across 10 different domains SCCHD-physician: 11 questions, seven which correspond with SCCHD Shared domains between two questionnaires: Treatment goals at diagnosis; Quality of life; EOL decision making; Quality of communication and caregiver-family relationship Time phase = ‘time after which you realized your child had no realistic chance of survival’ and includes items focussing on last month of life	SCCHD: as above SCCHD-physician survey developed from SCCHD (further details not provided)	USA Single large paediatric cardiology centre (hospital)	Bereaved parents and primary cardiologists To children (<21 years) who died from any type of heart disease (Jan 2007–Dec 2009)	33/78 bereaved parents completed QA (42% RR); 30 (97%) female; mean age 47.4 year; 29 non-Hispanic white (94%) 31/33 cardiologists completed QA (94% RR); demographics N/S. Total = 31 parent/physician pairs. Representing 31 children; median age 6 month (range 4 days–20.4 year); gender and ethnicity N/S	No specific psychometric testing conducted	15% bereaved parents thought their child had suffered ‘a great deal’ while no cardiologist did 17 (55%) bereaved parents perceived they were unprepared for the way their child died c.f. 29% cardiologists; little agreement between 12/28 (43%) parent/physician pairs 29 (93%) bereaved parents perceived quality of care in last month was ‘excellent/very good’ compared with 24 (78%) cardiologists

EOL = end-of-life; ICU = Intensive Care Unit; N/S = not stated; OR = odds ratio; QA = questionnaire; QoL = quality of life; RR = response rate; USA = United States of America.

**Table 5.** Content of the tools mapped to the ‘good death of a child’ dimensions.^8^

Dimension	Participation	Personal style	Quality of life	Preparation for death	Aspects of care	Legacy	Impact on survivors	Other domains within tools
Examples of attributes of dimensions	Awareness of dying/acceptance; autonomy/timing/location (of death); expectations and personal ideal	Dignity; affirmation of whole person; individuality/personal/privacy	Pain and symptom management; social relations; survival goals	Advance care planning; honesty/communication; hope; completion	Aspects of staff; Continuity; Cultural and spiritual concerns	Having someone present; contributing to others; establishing meaning; importance of ritual/funeral	Grief resources; economic resources	
*Tool*								
GDI-P	Y	Y	Y	Y	Y	Y	N	N
Domains mapped to dimensions	A peaceful death in the presence of family	Living a normal life	Relief from physical and psychological suffering; Spending time with the family	Minimum medical treatment	Good relationships with medical staff	Making wonderful memories and fulfilling wishes; Playing and learning		
PICU-QODD-20	Y	Y	Y	Y	Y	Y	Y	Y
Domains (and specific question items where needed) mapped to dimensions	Privacy and PICU environment issues (item about parental privacy to be with child at end-of-life)	Emotional needs/support of family (item about clinical staff cared about ‘the child as an individual’)	Pain and symptom management; Emotional needs/support of family	Communication issues; Decisions to withdraw life support	Spirituality and religion/cultural issues; Continuity/coordination of care	Fulfilling the parental role	Grief and bereavement	Physical and instrumental needs of family (items about bathroom/carpark facilities)
GDI	Y	Y	Y	Y	Y	Y	N	Y
Domains mapped to dimensions (including eight optional domains)	Dying in a favourite place; Natural death; Unawareness of death	Being respected as an individual; Maintaining hope and pleasure; Independence; Pride and beauty	Physical and psychological comfort; Good relationships with family	Receiving enough treatment; Control over the future; Preparation for death	Good relationships with medical staff; Religious and spiritual comfort	Life completion; Not being a burden to others; Feeling that one’s life is worth living		Environmental comfort
FAMCARE*	N	N	Y	Y	Y	N	N	Y
Domains mapped to dimensions			Pain and symptom management	Satisfaction with communication with HCP	Availability of clinicians			Family satisfaction with cancer care
Toolkit**	Y	Y	Y	Y	Y	N/S	N/S	N/S
Question items mapped to dimensions (study only highlighted specific question items)	Item about ‘knew what to do at the time of death’	Item about how well ‘the patient died with dignity’	Items about how well ‘the patient’s symptoms were controlled’ and ‘providing emotional support’	Items about ‘was information given about what to expect about dying’ and ‘did doctors listen to concerns?’	Item about ‘spiritual/religion addressed?’			
SCCC	Y	N	Y	Y	Y	N	Y	N
Question items mapped to dimensions	Items about location and peacefulness of the child’s death		Items about symptoms and their treatment; quality of life and emotional well-being	Items about decision-making at the EOL (e.g. DNACPR); quality of care and communication	Items about degree of physician/home care team involvement in EOL care; teamwork; religious/spiritual mentor		Items about burdens after child’s death; contact after death; economic impact of child’s terminal illness	
SCCCH (study focus only on specific areas; so unable to state whether more dimensions covered)	N/S	N/S	Items about symptom control and quality of life	Items about communication with care team and use of treatment-directed technologies at EOL	N/S	N/S	N/S	N/S
PaPEQu**	Y	Y	Y	Y	Y	N/S	Y	N/S
Domains (and specific question items where needed) mapped to dimensions	Grief and bereavement support (item about ‘choosing the place of death’)	Holistic care of the child	Relief of pain and other symptoms; Support of the family unit	Involvement of child and family in communication, decision-making and care planning	Continuity of care; Support of the family unit (item about access to ‘spiritual counselling’)		Grief and bereavement support	
EXPERIENCE @Home Measure (final 22 items)	Y	Y	Y	Y	Y	N	N	Y
Question items mapped to dimensions	Item about ‘last weeks of life and what they may be like’	Item about ‘care team considers all of my child’s needs’	Items about child’s physical symptoms and emotional support; support of parent; sibling support	Items about decision-making, information provision, trust, hope	Items about coordination of care, knowledge and skills of healthcare team			Items about on-call services and adaptation of home
Quality of Children’s End-of-life Care Instrument	Y	Y	Y	Y	Y	N	Y	Y
Domains (and specific question items where needed) mapped to dimensions	Provide care at death	Connect with families (item included being treated ‘as a unique person’)	Support parents; Support the child (items about physical, emotional, social and spiritual needs); Support siblings	Share information with parents; Involve parents	Share information among HCP; Connect with families; (items about spiritual needs and cultural/spiritual/religious practices asked within three separate domains)		Provide bereavement follow-up	Structures of care (items include food and car parking)
PICU-QODD	Y	Y	Y	Y	Y	Y	Y	N
Question items mapped to dimensions	Items about feeling at peace with dying, saying goodbye, being present at moment of death	Items about keeping dignity and self-respect	Items about pain, breathing, spending time with family/friends	Items about receiving support from ventilator, discussing wishes for end-of-life care	Items about visits from religious/spiritual leader, having spiritual service/ceremony and care received from healthcare team	Items about making end-of-life plans or funeral arrangements	Items about healthcare costs	

Y: yes; N: No; N/S: not stated (detail not provided within study); DNACPR=do not attempt cardio-pulmonary resuscitations; EOL: end-of-life; HCP: healthcare professional; PICU: paediatric intensive care unit.

* Only communication items were reported within study; further information about FAMCARE items obtained from http://www.npcrc.org/files/news/famcare_scale.pdf.

** Full details of question items used not provided within study and did not receive response from corresponding author.

With the exception of one tool,^35^ all the other tools were developed for use after death. The time period in which the child’s death had occurred ranged from within a previous 12-month period^25^ up to the previous 7 years.^28^ No tool had been developed or used directly with patients (child or young person) during the dying phase of their illness nor specifically with siblings. The definitions of the specified assessment period varied and could include the last 3 days (*n* = 2),^24,25^ last 4 weeks (*n* = 1)^33^ or last month prior to death (*n* = 1).^28^ Additionally, the phrase ‘the time before death when the physician estimated that the child had no realistic chance for cure’ was used (*n* = 2).^30,40^ For the remaining tools, the assessment period wasn’t defined within the study, but question items specifically asked about dying or death. Most (*n* = 10) tools were used within the context of a survey; the other, had also been used within an interview setting (face-to-face or via telephone).^28^ One of the surveys was undertaken alongside a concurrent qualitative interview.^38^

All the tools assessed aspects of quality of life (e.g. pain and symptom control) and preparation for death (e.g. communication, decision-making). Items relating to ‘legacy’ (e.g. establishing meaning, importance of ritual/funeral), were assessed within five tools (Table 5). Question items less frequently asked about cultural aspects of care (*n* = 2), economic costs (*n* = 2) and grief and bereavement (*n* = 4).

### Tools used predominately with healthcare professionals

The two tools used with healthcare professionals were the Good Death Inventory – Paediatrics (GDI-P)^23,24^ and the Paediatric Intensive Care Unit – Quality of Dying and Death 20 (PICU-QODD)^25^ (Table 1).

#### Quality of tool

Both tools underwent a robust process of development and have been tested for validity and reliability.^23,24^ PICU-QODD-20 has question items mapping across all seven dimensions of a ‘good death’.^25^

#### Clinical implications

Whereas the GDI-P purpose is focussed on nursing perspectives of paediatric cancer deaths across several care settings (including hospital, PICU and home),^23^ the PICU-QODD-20 seeks to obtain a variety of healthcare professional perspectives about deaths due to different illnesses but only for those occurring in PICU.^25^

### Tool used solely within a cancer population

The four tools used solely within a cancer population were: Good Death Inventory (GDI),^26^ Family Satisfaction with the End-of-Life Care (FAMCARE),^27^ the Toolkit After-Death Bereaved Family Member Interview (subsequently referred to as the ‘Toolkit’)^27^ and a questionnaire, initially developed by Wolfe et al.,^28^ which was later called Survey about Caring for Children with Cancer (SCCC)^29–31^ (Table 2).

#### Quality of tool

The SCCC is the most extensive tool (211 items)^28–31^ with question items spanning across many different aspects of cancer care as well those relating to care at the very end of life. It has undergone a careful process of question item development and selection. FAMCARE and the ‘Toolkit’^27^ are established, validated tools previously used with bereaved families for adult deaths. Only the GDI,^26^ however, has undergone initial psychometric testing of validity and reliability specifically within a palliative paediatric population. None of the tools incorporated all aspects of multi-dimensionality in terms of a ‘good death’.

#### Clinical findings

Findings from the study using the GDI indicated that aspects of advance care planning (e.g. establishing a ‘living will’) were associated with more positive parental perspectives about a ‘good death’.^26^ Both FAMCARE and the ‘Toolkit’ were used within the same study, assessing the quality of end-of-life care for adolescents and young people (aged 15–39 years) from the caregiver perspective.^27^ The study showed most caregivers were satisfied with care, but there were unmet information and religious/spiritual care needs.^27^ SCCC has been used within four studies conducted in two different countries. Within the first study, 92 (89%) bereaved parents reported their child experienced ‘a lot’ or ‘a great deal’ of suffering’, although 70% said the actual death was ‘very peaceful’.^28^ A further study found that those receiving home care services were more likely to die at home.^29^ An additional two studies, conducted within a single state in Germany, enabled a comparison of quality of end-of-life care over two time periods.^30,31^ Although symptom reporting was similar, preferences about place of death were more concurrent with actual place of death in the second study.^31^

### Tool used with both cancer and non-cancer populations

The four tools used within both cancer and non-cancer populations were: the PELICAN questionnaire (PaPEQu) ^32,33^; the Experience @HOME Measure ^35,36^; the Quality of Children’s End-of-life Care Instrument ^37^ and the PICU-QODD^38^(Table 3).

#### Quality of tool

The first three tools have all undergone a robust process of development ^32,35,37^; the PICU-QODD was modified from an existing, validated tool used with bereaved families for adult deaths.^38^ All tools except the Experience @HOME Measure have reported on their psychometric properties with the PaPEQu being the most extensively reported.^32^ Only the PICU-QODD covers all seven dimensions of a ‘good death’.^38^

#### Clinical findings

The Experience @HOME Measure focuses purely on the home care setting. It is the only tool intended to be used before death and retrospectively assesses care provided in the previous week.^35,36^ The Quality of Children’s End-of-life Care Instrument focuses on the bereaved mothers’ perspective of the quality of end-of-life care.^37^ Both the PaPEQU and the PICU-QODD have been used within clinical studies. PaPEQU has been used to assess quality of end-of-life care for children who died from a variety of illnesses (cardiac, neurological or oncological illness or during the first 4 weeks of life).^32^ Studies show that bereaved parents’ perceptions about overall care were highest for children dying with cancer, those who had engaged with Paediatric Palliative Care teams, and lowest for children dying with neurological conditions or in the neonatal period.^32,33^ The PICU-QODD was used alongside a qualitative interview and explored both bereaved parents and grandparents’ views about end-of-life care. The majority of aspects of care within the PICU-QODD were rated highly, whereas the qualitative findings highlighted the need for more direct communication with healthcare professionals.^38^

### Tool used solely within a life-limiting cardiac population

The one tool used within a life-limiting cardiac population is the Survey for Caring for Children with Advanced Heart Disease (SCCHD)^39,40^ (Table 4).

#### Quality of tool

This was developed from the Wolfe et al.^28^ questionnaire,^39^ although no psychometric testing has been reported.

#### Clinical findings

A subsequent study used the SCCHD to assess both bereaved parents and cardiologist views reflecting different perspectives about the degree of preparation for death and overall quality of care.^40^

## Discussion

### Main findings

This scoping review identified 11 tools, developed and used across seven countries, which assess the quality of dying, death and end-of-life care for children and young people. The majority of tools have been used after the child’s death with bereaved parents, predominantly mothers, in a hospital setting. In terms of content, all tools asked about quality of life and preparation for death whereas aspects relating to cultural concerns, financial costs, grief and bereavement were more variable. The PICU-QODD-20 and PICU-QODD had the most comprehensive content across the dimensions of a ‘good death’.

Only six tools have undergone some degree of psychometric testing for validity and reliability specifically within a paediatric palliative care population. Those which have reported the most extensive testing for validity and reliability are GDI-P, PICU-QODD-20 and PaPEQu, whereas initial findings were more limited for the GDI, the Quality of Children’s End-of-Life Care Instrument and PICU-QODD. Although the SCCC has not undergone formal psychometric validation, it represents an extensive ‘question bank’ which has been developed and used across two different countries to assess quality of end-of-life care. No tool has addressed the challenges of assessing the views of children or young people themselves or specifically been used to assess the perspective of siblings.

### What this study adds

Whilst previous systematic reviews, have focussed on health-related quality of life outcome measures,^11^ none have been directed towards identifying tools used to assess quality of care provided at the end of a child’s life. This scoping review allows comparison of tools and helps identify gaps for which future research is needed.

Establishing whether the identified tools are suitable for use in a wider cultural context is required. Existing studies have predominately been undertaken within the USA, which has a specific type of healthcare system, reliant on health coverage and economic resources. No tools have been developed or revised to be used within the UK, Ireland, Canada nor Australia, which are all regarded as having a high level of palliative care integration into mainstream children’s healthcare services.^41^ The majority of studies were conducted, at least in part, within hospital settings. This may reflect specific cultures such as that within the UK, where most children and young people’s deaths occur in hospital.^42^ International partnerships have previously been recommended to enhance learning and inform tool validation.^43^ Hence, there is a need to establish whether existing tools are relevant and meaningful across much more diverse countries and cultures. This is especially pertinent when terms such as ‘grief’ and ‘distress’ can be specific to the English language.^44,45^

Rather than developing new tools, future focus should be on further improving and validating existing tools. It is also important to consider whether the identified tools have utility within different clinical settings. For example, the content of PICU-QODD-20 covered all seven dimensions of a ‘good death’ and has been assessed for some aspects of validity and reliability.^25^ The remit of the tool, however, is within a very specific intensive care environment. It would be important to establish whether this tool could be adapted and have wider application. The SCCHD offered comparative views about care from both the bereaved parents and the cardiologists’ perspective.^40^ As there are two different versions of the GDI and the PICU-QODD (one for healthcare professionals; one for bereaved parents),^24–26,38^ these tools also offer that possibility. Establishing whether tools such as these could be adapted to incorporate the views of siblings would also be of value. The Experience @HOME Measure is the only tool used before death.^35^ Hence, exploring the possibility of the dying child’s ability to participate in completion would be a further area of exploration.

Only one study combined the use of a tool with an individual qualitative interview.^38^ The opportunity for bereaved relatives to be able to ‘tell their story’, to share narrative accounts, is recognized to have potential therapeutic benefit.^46^ Hence, it would seem important for existing tools to include free-text space to enable opportunities for sharing experiences not captured within the specific question domains. Additionally, it has been recognized that there is strength in combining both qualitative and quantitative approaches for paediatric palliative care research^47^ – evaluation of quality of dying, death and end-of-life care would be an area where both rigorously developed outcomes and qualitative approaches would enrich the detail of reported experiences.

### Strengths and limitations of the study

The search strategy conducted followed a robust, systematic methodology and included grey literature, reverse citation searching and screening of reference lists. We were not able to contact every individual author to enquire about additional work/unpublished studies, hence some relevant studies may have been overlooked. Additionally, our main focus was on the identification and development of available tools so subsequent studies focussing only on their use, may have been omitted. In keeping with the aims of a scoping review, we did not undertake a formal assessment of study quality nor psychometric properties. As the reporting of these details within each study was not always consistent, there may be some ambiguity when directly comparing different tools. Additionally, we did not consider all the principles which can be used to assess quality-of-life instruments for example respondent and administrative burden. The choice of our dimensions for a ‘good death’ came from a study which, although involved multiple stakeholders, was focussed on children dying from cancer.^9^ Experiences about what constitutes a ‘good death’, however, is complex and multi-faceted, potentially varying for different types of life-limiting illnesses.^48,49^

## Conclusion

This review has identified 11 available tools for assessing quality of dying, death and end-of-life care in paediatrics, yet there is variability in terms of instrument content and evidenced quality (i.e. degree of assessment of validity and reliability). Improvement of existing tools should involve the inclusion of additional items representing salient domains of a ‘good death’ and further psychometric testing to ensure more valid, reliable and comprehensive assessment. International partnerships are key to determining suitability for wider use, informing tool validation and application across different countries and cultures. Despite the recognized challenges, sensitive and timely ways to identify data about the last weeks of life, can help facilitate learning about experiences, leading to further improvements in quality of care both before and after the death.

## Supplemental Material

sj-docx-1-pmj-10.1177_02692163221105599 – Supplemental material for Measuring quality of dying, death and end-of-life care for children and young people: A scoping review of available tools Click here for additional data file. Supplemental material, sj-docx-1-pmj-10.1177_02692163221105599 for Measuring quality of dying, death and end-of-life care for children and young people: A scoping review of available tools by Catriona R Mayland, Katy A Sunderland, Matthew Cooper, Paul Taylor, Philip A Powell, Lucy Zeigler, Vicki Cox, Constance Gilman, Nicola Turner, Kate Flemming and Lorna K Fraser in Palliative Medicine
