# Supplementary material for: Measuring quality of dying, death and end-of-life care for children and young people: A scoping review of available tools
Source: Palliat Med. 2022 Aug 1;36(8):1186–206. doi: 10.1177/02692163221105599 (PMC9446433; doi:10.1177/02692163221105599)
Supplement: sj-docx-1-pmj-10.1177_02692163221105599 – Supplemental material for Measuring quality of dying, death and end-of-life care for children and young people: A scoping review of available tools [file sj-docx-1-pmj-10.1177_02692163221105599.docx]

| Supplementary file 1. Search Strategy for CINAHL Database |
| --- |
| 1. (“Quality of dying”).ti,ab 2. (“Quality of death”).ti,ab 3. (“good death”).ti,ab 4. (“bad death”).ti,ab 5. (quality).ti,ab 6. (“end of life”).ti,ab 7. “ATTITUDE TO DEATH”/ 8. (tool).ti,ab 9. (measur*).ti,ab 10. (scale).ti,ab 11. (instru*).ti,ab 12. (assess*).ti,ab 13. (question*).ti,ab 14. (survey).ti,ab 15. (questionnaire).ti,ab 16. (test).ti,ab 17. (outcome*).ti,ab 18. exp QUESTIONNAIRES/ OR exp “OPEN-ENDED QUESTIONNAIRES”/ 19. exp “TERMINALLY ILL PATIENTS”/ 20. exp RESUSCITATION 21. exp “LIVING WILLS”/ 22. “TERMINAL CARE”/ OR “HOSPICE CARE”/ OR “PALLIATIVE CARE”/ OR “RESUSCITATION ORDERS”/ 23. “HOSPICE AND PALLIATIVE NURSING”/ 24. ((terminal* OR advanced OR incurable OR life-limit* OR life-threaten*) ADJ2 (ill* OR disease* OR condition* OR stage*)).ti,ab 25. (terminal* ADJ2 (care OR caring)).ti,ab 26. (end ADJ2 life).ti,ab 27. (palliat*).ti,ab 28. (hospice*).ti,ab 29. (dying).ti,ab 30. ADOLESCENCE/ OR “ADOLESCENT, HOSPITALIZED”/ OR CHILD/ OR “MINORS (LEGAL)”/ 31. INFANT/ OR “INFANT, DRUG-EXPOSED”/ OR “INFANT, HIGH RISK”/ OR “INFANT, HOSPITALIZED”/ 32. PEDIATRICS/ 33. (adolescen* OR teen* OR youth* OR juvenile* OR minors OR child* OR schoolchild* OR preschool* OR toddler* OR boy* OR girl* OR paediatric* OR pediatric* OR infant* OR infancy).ti,ab 34. (5 AND 6) 35. (1 OR 2 OR 3 OR 4 OR 7 OR 34) 36. (8 OR 9 OR 10 OR 11 OR 12 OR 13 OR 14 OR 15 OR 16 OR 17 OR 18) 37. (19 OR 20 OR 21 OR 22 OR 23 OR 24 OR 25 OR 26 OR 27 OR 28 OR 29) 38. (30 OR 31 OR 32 OR 33) 39. (35 AND 36 ANS 37 AND 38) 40. 39[DT 2000-2021][Languages eng] |
